# Group-based exercice training programs for military members presenting musculoskeletal disorders – protocol for a pragmatic randomized controlled trial

**DOI:** 10.1186/s12891-022-05317-6

**Published:** 2022-04-18

**Authors:** F. Dupuis, K. Perreault, L. J. Hébert, M. Perron, Maj A. Fredette, F. Desmeules, J. S. Roy

**Affiliations:** 1 Center for Interdisciplinary Research in Rehabilitation and Social Integration (Cirris), Québec, Canada; 2 Département de réadaptation, Faculté de médecine, Université Laval, Québec, Canada; 3 Département de radiologie et médecine nucléaire, Faculté de médecine, Université Laval, Québec, Canada; 4 Canadian Armed Forces, BFC USS Valcartier, Québec, Canada; 5 École de réadaptation, Faculté de médecine, Université de Montréal, Montréal, Canada; 6 Centre de recherche de l’Hôpital Maisonneuve-Rosemont (CRHMR), Montréal, Canada

**Keywords:** Musculoskeletal disorders, Low back pain, Rotator cuff related pain, Ankle sprains, Patelofemoral pain syndrome, Physiotherapy, Group-based intervention, Individual intervention, Randomized controlled trial

## Abstract

**Background:**

Musculoskeletal disorders are a leading cause of morbidity and the most prevalent source of disability among soldiers. Their high prevalence in armed forces and limited ressources have led to problems related to access to physical rehabilitation care. To increase access, supervised group-based exercise programs for the most prevalent musculoskeletal disorders (low back pain, patellofemoral pain, rotator cuff-related shoulder pain or lateral ankle sprain) have been developed at a Canadian Armed forces (CAF) base, but their effectiveness has not been evaluated. The primary objective of this randomized controlled trial is to evaluate the mid- and long-term effects of these group-based training programs on pain severity and functional limitations, in comparison with usual individual physiotherapy care. Secondary objectives include comparing both interventions in terms of health-related quality of life, pain-related fear, and patients’ satisfaction.

**Methods:**

One hundred and twenty soldiers with a new medical referral for physiotherapy services for one of the four targeted musculoskeletal disorders will be consecutively recruited. They will be randomly assigned to either group-based training program or usual individual physiotherapy care, and will take part in the assigned 12-week intervention. There will be four evaluation sessions over 26 weeks (baseline, week 6, 12 and 26). At each follow-up, functional limitations, pain severity, health-related quality of life and pain-related fears will be assessed. Patients satisfaction with treatment will also be evaluated at the end of the intervention period. Either two-way repeated measures ANOVA will be used to analyse and compare the effects of the interventions.

**Discussion:**

This RCT will determine the effectiveness of group-based training programs compared to usual individual physiotherapy care. This new intervention model could represent an efficient, and more pro-active approach to manage a higher number of soldiers with musculoskeletal disorders. It could improve access to physical rehabilitation care and improve the health of soldiers.

**Trial registration:**

ClinicalTrials.gov (NCT05235152), February 11th 2022.

## Background

Musculoskeletal disorders (MSKd) affect 20 to 33% of the world’s population [1, 2]. Due to their great prevalence and their important consequences on physical and mental health [3], MSKd are the leading cause of loss of productivity and invalidity worldwide [2]. Conservative management, including rehabilitation interventions such as education and exercises, is well recognized as a first choice therapeutic option for persons presenting these disorders [4]. However, limited access to such outpatient physical rehabilitation services has been identified as a major problem in many countries [5–8]. Access to these services is notably limited by long wait times, leading to increased disability and invalidity [3, 9].

Members of Armed Forces are among those affected by extensive waiting times to access physical rehabilitation services [10]. MSKd are a leading cause of morbidity and the most prevalent source of disability among military personnel [11–13]. They also represent the main medical cause of restricted duties and days off work, and the main reason for not being able to deploy [13, 14]. In the last decade, the increased operational tempo, the aging of soldiers, and the changing nature of the missions have led to a higher number of non-battle-related MSKd [14, 15]. The management of MSKd in a timely manner has thus become a huge challenge.

Physical rehabilitation among military members with MSKd requires innovative effective interventions to optimize the use of ressources and increase access to these services. As the literature encourages the use of active exercises for the management of MSKd [4, 16], the development of a tailored supervised group-based exercise approach could represent an option of choice. Supervised group-based exercise programs have been shown to reduce wait times to rehabilitation services [17] and to be effective in the management of MSKd [18]. However, the majority of studies until now have evaluated group-based interventions for chronic low back pain [18]. In the last 10 years, three supervised group-based exercise programs supervised by physiotherapists (PT) and physiotherapy technologists (Phys. T.) have been developed at one of the Canadian Armed forces (CAF) bases (Valcartier) (Lumbar Training Program for for low back pain; Upper Extremity Training Program for rotator cuff-related shoulder pain; Lower Extremity Training Program for patellofemoral pain syndrome and lateral ankle sprain). They were developped to increase access to physical rehabilitation care regarding the most prevalent MSKd, as CAF statistics show the lumbar spine, knee, ankle and shoulder are by far the most frequently affected body parts, contributing to 58.2% of all cases of MSKd [15, 19–21]. Although these group-based exercise programs have been widely used, their clinical effectiveness needs to be evaluated in large samples or compared to usual individual physiotherapy care (UPC).

The primary objective of this pragmatic randomized controlled trial [22] is to evaluate the mid- and long-term effects of group-based supervised training programs on pain severity and functional limitations, in comparison with UPC in the treatment of military members presenting common MSKd. Secondary objectives include comparing both interventions 1) in terms of health-related quality of life, 2) pain-related fear, and 3) satisfaction with care received. We hypothesize that the group-based supervised training programs will be at least as effective as UPC to decrease functional limitations and pain severity, to improve health-related quality of life and pain-related fears and participants will be equally satisfied with both interventions.

## Methods

### Participants

All military members with a new medical referral for physiotherapy services at the Valcartier Health Centre for either low back pain, patellofemoral pain syndrome, rotator cuff-related shoulder pain or lateral ankle sprain and who agree to participate in the study will be consecutively recruited. A PT will evaluate all potential participants to confirm their eligibility. To be eligible, military members will have to be aged between 18 and 60 years and present one of the four targeted MKSd:

Low back pain: Inclusion – 1) LBP with or without radiation to the lower limbs, 2) minimal score of 17% on the Modified Oswestry Disability Index (ODI) (based on its clinically important difference [CID] [23]). Exclusion – 1) history of surgery or facture to the spinal column, 2) signs of upper motor neuron lesions (bilateral paresthesia, hyperreflexia or spasticity) [24] or other redflags (e.g. fracture) [25].

Patellofemoral pain syndrome: Inclusion – 1) anterior knee pain during running or during at least two activities among: kneeling, squatting, and resisted knee extension [26], 2) score lower than 85/100 on the Knee Outcomes Survey – Activity of Daily Living Scale (KOS-ADLS) [27]. Exclusion – 1) history of knee surgery or patellar dislocation; 2) pain believed to originate either from meniscus (presence of joint line fullness and tenderness, McMurray sign, and positive Thessaly test) [28] or from any knee ligament.

Rotator cuff-related shoulder pain: Inclusion – 1) at least one positive finding in each of the following categories: a) painful arc of movement; b) positive Neer’s or Kennedy-Hawkins Test; c) pain on resisted external rotation, resisted abduction or Empty Can Test [29], 2) minimal score of 14 points on the *Quick*DASH [30]. Exclusion – 1) history of shoulder surgery, fracture, capsulitis, or dislocation, 2) full thickness rotator cuff tear identified by imagery or clinical tests [31], 3) cervicobrachialgia or shoulder pain reproduced by neck movements.

Lateral ankle sprain: Inclusion – 1) unilateral lateral ankle sprain of < 6 weeks, 2) minimal score of 9 points on the LEFS [32]. Exclusion – 1) ankle fracture, 2) lateral ligaments not the principal injury (a high ankle/tibiofibular sprain).

For all four MSKd, potential participants will be excluded if they 1) are unavailable to participate in a 12-week intervention; 2) have a diagnosis of rheumatoid, inflammatory, neurological or neurodegenerative disease; 3) received a corticosteroid injection in the previous 6 weeks in the affected region; or 4) have had more than 6 months of work restriction for their current MSKd [20]. Furthermore, patients admitted to the physiotherapy clinic of the Valcartier Health Centre with an acute condition and who present constant and intense pain (> 5/10), severely limited range of motion (more than 50% in at least 2 directions) [20], obvious lateral shift for LPB or unable to bear weight (for lateral ankle sprain) will first be treated one-on-one by a PT and then be referred to the research team once the condition is no longer acute, based on the same criteria. Acute conditions that require one-on-one follow-up initially will be documented.

### Sample size

Based on our sample size calculation, calculated for our primary outcome (Pain Interference subscale of the Brief Pain Inventory) using data from a pilot study on military members with MSKd [33], 15 participants per diagnosis will be required per group for a total of 120 participants (60 participants/group) (G*Power 3.1.7; α = 0.05, 1-β = 0.95, Baseline mean: 2.9+/− 1.5 points, Follow-up mean: 0.8+/− 1.2 points, expected lost at follow-up = 15% [based on past experiences with this population]. This number will enable us to determine the effects of the interventions for each of the four conditions.

### Study design and experimental procedures

**Fig. 1:**
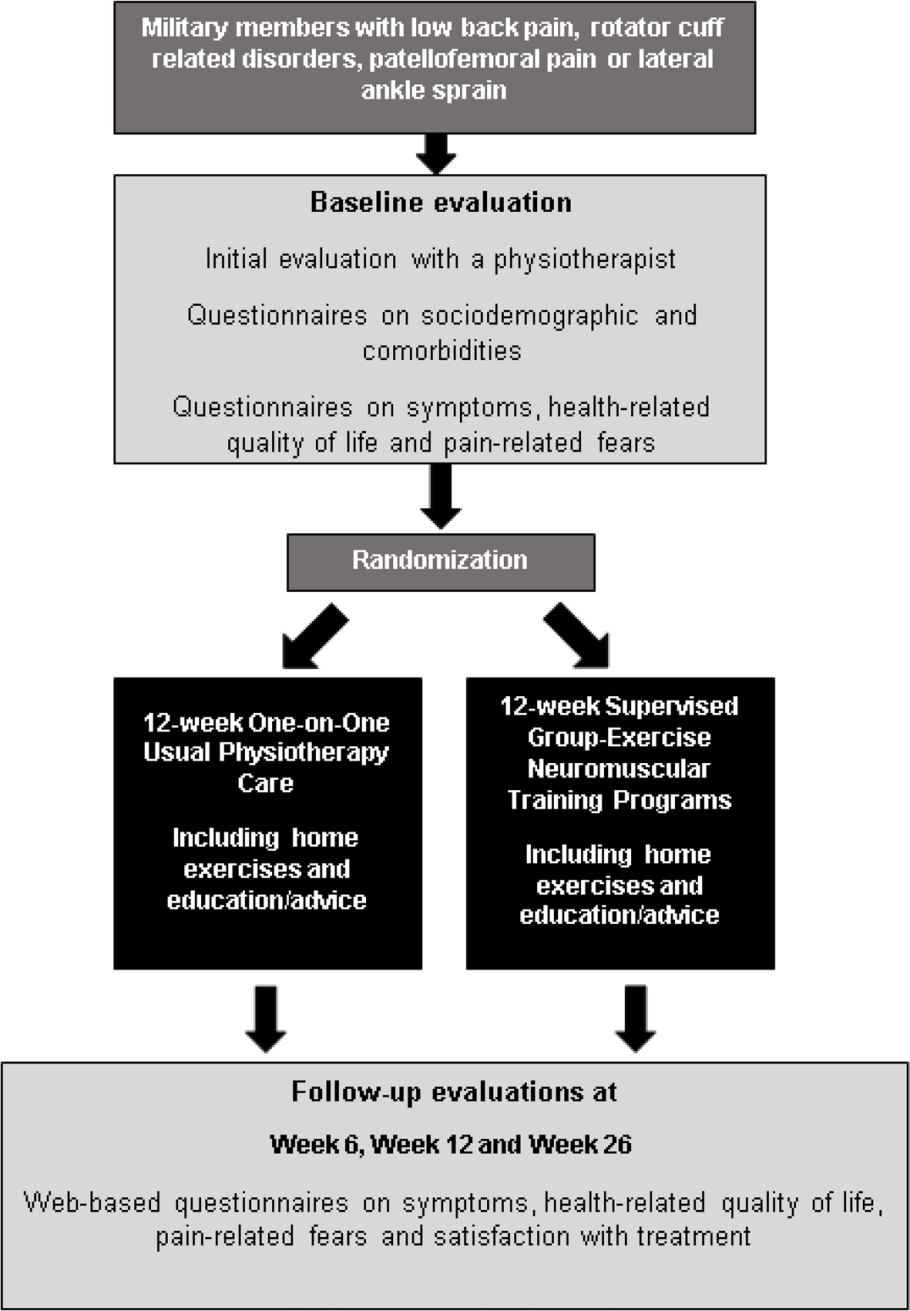
Study design

This pragmatic parallel-group RCT will compare two 12-week interventions and includes four evaluation sessions over 26 weeks (baseline, week 6 [mid-intervention], week 12 [end of intervention] and week 26). Once the written informed consent form will have been signed and eligibility criteria confirmed by an independant PT, participants will take part in the baseline evaluation. At baseline, participants will first complete a questionnaire on sociodemographic, symptoms and comorbidity. Then, they will be randomly assigned to either the 1) group-based supervised training program or 2) usual individual physiotherapy care. They will then take part in their assigned 12-week intervention at the Valcartier Health Centre (Fig. 1). At follow-up evaluations, the primary and secondary outcomes will be assessed using web-based questionnaires integrated within the Research Electronic Data Capture platform (REDCap) [34]. This pragmatic randomized controlled trial protocol have bee registered on clinicaltrials.gov (NCT05235152) [35].

### Randomisation/blinding

A randomisation list will be established using a computer random number generator (sealedenvelope.com) prior to the initiation of the study by an independent research assistant. Allocation will be concealed in sealed and sequentially numbered opaque envelopes. Randomisation will be stratified by sex and type of MSKd to ensure balance of the treatment groups with respect to these variables. A blocked randomisation (random blocks of 3 or 6) will also be used to make sure that two equal groups of 60 participants (15 participants/diagnosis) will be obtained. Given that for this type of intervention it is impossible to blind the treating PT and participants, only the evaluator and statistician will be blinded. To reduce potential contamination bias, the two interventions will be given in different closed rooms at the Valcartier Health Centre by different PTs. Participants will be instructed to avoid discussing their group assignment or interventions with other participants or with the evaluator.

### Interventions

Before randomization, all participants will be evaluated by a PT (i.e., individual evaluation) [36, 37]. Then, participants will be randomized and will take part in their assigned 12-week intervention, which will consist of a maximum of 16 in-person sessions of 30 to 45 min each. An individualized home exercise program lasting 20–30 min, to be performed 3–4 times per week, will also be given to the participants in both groups.

If participants are symptom-free or do not have any functional limitations left before the end of the 16 sessions or 12 weeks, treatment will be ceased as judged by the PT supervising the group-based interventions or the individual treatment. The home exercises program will then be continued, with the possibility to resume the intervention if there is a recurrence of pain or functional limitations within the 12-week intervention period.

#### Usual individual physiotherapy care (UPC)

PTs will be asked to provide UPC that best reflects usual physiotherapy interventions at the physiotherapy clinic of the Valcartier Health Centre, that is an education-based (e.g., physiopathology, pain management, activity modification, reassurance, basic pain neuroscience, pain related fears, pain catastrophizing) [38], active exercise rehabilitation approach which includes strengthening and neuromuscular training exercises (including proprioception). In addition, interventions targeting mobility (stretching; active, active-assisted, passive range of motion exercises; repeated movements [Mulligan or McKenzie]) and manual therapy will be used as recommended in clinical practice guidelines [36, 37]. Patients will thus receive the treatments they would normally receive, and interventions will be selected according to PT’s and patients’ preferences.

#### Group-based supervised training program

The low back pain, upper extremity and lower extrimity Training Programs were developed for military members with MSKd by the Valcartier Health Centre PTs based on a literature review and clinical experience [20, 21]. These group-based training programs have been implemented and used for several years in addition to individual physiotherapy to increase the number of patients managed. They are composed of stations that each include several exercices of varying levels of difficulty (see further description below). Group size will vary between 5 to 20 participants for one PT or PT technologist, and each military member performs his/her own exercices. For example, during a typical session, the participant and PT will choose one exercise to perform per station according to two main criteria: severity of the condition (i.e., symptoms and limitations) and the ability to perform the exercises optimally. The level of supervision will be adapted to the participant’s needs and performance. Exercises are progressed if the quality of movement control is considered adequate by the PT and the exercise is no longer a challenge according to the participant. Exercise parameters and progressions of the programs have been chosen to increase strength, endurance and neuromuscular control [39, 40]. Progression in the programs leads to the execution of exercises that simulate functional and occupational tasks (task-oriented approach with gradual exposure) [21]. In accordance with recognized motor learning principles, participants will be encouraged to complete a large variety of exercises and to focus on the quality, rather than the quantity of their movements [41]. Exercises will be paused if pain is greater than 3/10 during execution or if fatigue results in compensatory movements [42].

The Lumbar Training Program is composed of 7 stations. The exercises are grouped together as follows: hip strengthening and control; the squat and its variants; elastic bands and the body blade; abdominal planks and their variants; abdominal strengthening; back extensor strengthening; and lifting techniques [20]. The Upper Extremity Training Program for rotator cuff-related shoulder pain is composed of 10 stations. The stations include postural and scapulothoracic control; weight bearing exercises; neuromuscular re-education of the rotator cuff complex; serratus anterior strengthening; trapezius strengthening; body blade exercises; proprioception and motor control exercises; functional activities; push-ups, bench press and sand bad lift [21]. The Lower Extremity Training Program for patients with pattelofemoral pain syndrome and lateral anke sprain is also composed of 10 stations. The stations include static one-leg balance; hip control exercises; jump landing; BOSU exercises (jumping consecutively on top of, and then on the other side of the BOSU); one-leg side jumping; jumping on stones; trampoline exercises; stability exercises; static and dynamic balance exercises on a beam; dynamic control exercises [43–45].

### Outcomes

The primary outcome will be functional limitations, as measured with the Pain Interference subscale of the Brief Pain Inventory – Short Form (BPI). The secondary outcomes will include pain severity as measured with the Pain Severity subscale of the BPI, and health-related quality of life as measured with the EQ-5D-5L. As pain-related fear has been shown to influence outcomes in military members and conservative management reduces pain-related fears [20, 46], both interventions will also be compared on their impact on pain-related fears, measured with the Tampa Scale for Kinesiophobia (TSK) [38]. Furthermore, region-specific symptoms and functional limitations will be assessed using the Oswestry Disability Index (ODI) for low back pain, the shortened version of the Disabilities of the Arm, Shoulder and Hand (*Quick*DASH) for rotator cuff-related shoulder pain, Knee Outcome Survey Activities of Daily Living Scale (KOS-ADLS) for patellofemoral pain syndrome and Lower Extremity Functional Scale (LEFS) for lateral ankle sprain. To monitor compliance with home exercises, participants will be asked to maintain a weekly web-based logbook on RedCap during the 12 weeks of the intervention. Finally, participant’s satisfaction with treatment will be assessed at the end of the treatment (i.e., week-12) (see bellow for further description).

#### Functional limitations

The BPI is an 11-item questionnaire designed to evaluate the intensity of, and the impairment caused by pain. Originally developed to evaluate cancer pain, the BPI has since been shown to be a valid, reliable and responsive instrument for MSKd [47–50]. The Pain Interference subscale is recommended for assessment of pain-related functionnal limitations and includes seven items that measure the level of interference with function caused by pain using 0 (no interference) to 10 (complete interference) rating scales [49, 50].

### Pain severity

The Pain Severity subscale of the BPI includes four items that measure pain intensity using 0 (no pain) to 10 (pain as bad as you can imagine) rating scales [51].

### Health-related quality of life

The EQ-5D-5L is a generic health-related quality of life (HRQoL) questionnaire that contains five questions covering five dimensions: mobility, self-care, usual activities, pain/discomfort and anxiety/depression. Each question is rated on a five-point scale from 1 (no problems) to 5 (unable to perform). The combined dimensions describe 5^5^ = 3125 theoretically possible states of health that can be converted into a weighted index score ranging from 0 to 1. Its validity, reliability and responsiveness (CID = 0.32 point) have been established for MSK conditions [52, 53].

### Pain-related fear

The Tampa scale of kinesiophobia (TSK) is a 11-item scale measuring beliefs and behaviours related with pain, specially focusing on beliefs that pain is damaging and painful movements should be avoided [42]. Its psychometric properties have been shown for different MSKd [43].

### Region-specific symptoms and functional limitations

#### For low back pain

The modified ODI is a 10-item questionnaire that assesses the interference of low back pain with activities of daily living. Its reliability (MDC = 10 points), construct validity and responsiveness to change (CID ranges from 10 to 14) have been demonstrated [23, 54–57].

#### For patellofemoral pain syndrome

The KOS-ADLS (MDC90 = 8.3 points; CID = 13.6 points) is a 14-item knee-specific questionnaire that evaluates symptoms and functional limitations experienced during activities of daily living in individuals with various knee disorders [58].

#### For rotator cuff-related shoulder pain

The *Quick*DASH (MDC = 11%, CID = 10 points) is an 11-item questionnaire addressing the level of difficulty in performing daily activities and the severity of the symptoms of the upper limbs [59]. It has been validated in individuals with rotator cuff-related shoulder pain [30].

#### For lateral ankle sprain

The LEFS is a 20-item questionnaire assessing the impairment of the lower-extremity MSK system in everyday activities [60]. It has been validated in individuals with lateral ankle sprain, and its reliability, construct validity and responsiveness have all been demonstrated (MDC and CID are both 6 points) [61].

### Satisfaction

Patients satisfaction with treatment will be assessed at 6 and 12 weeks using the Patient Acceptable Symptom State (PASS) [62, 63], It asks patients weither they are satisfied with their current state or not and to rate their satisfaction on a 0–10 numeric scale (0 representing not satisfied at all, and 10 very much satisfied) [49]. They will also be asked to rate their satisfaction with treatment received using a three-item Likert scale (“not satisfied”, “satisfied” or “very much satisfied”), their satisfaction with the frequence of treatments (“not enough”, “just right” or “too much”), their satisfaction with the duration of treatments (“too short”, “long enough”or “too long”) and their satisfaction with the time spent with the PT during the treatments (“not enough”, “just right” or “too much”) [49, 51, 64].

### Statistical analyses

Descriptive statistics will be used for all outcome variables at each measurement time to summarise results. Baseline demographic data will be compared (independent *t*-tests and Chi-squared tests) to establish the comparability of groups. An intention-to-treat analysis will be used in which all participants will be analysed in the group to which they were assigned. In addition, sensitivity analyses will also be performed using per-protocol analyses; these analyses will allow to verify the consistency of the results in participants strongly compliant with the interventions (e.g., 75% attendance and home exercises). All dropouts and the reason for dropping out of the study will be reported. Any harm or unintended effects and co-interventions will be recorded. A 2-way ANOVA (2 Interventions [Group-Based or UPC] × 4 Times [0, 6, 12, 26 weeks]) will be used to analyse and compare the effects of the interventions on the primary outcome (Pain Interference Scale) and on four of the secondary outcomes (Pain intensity subscale, EQ-5D-5L, TSK) (SPSS, proc. GENLIN [Generalized Estimating Equations – GEEs]). Effect sizes (Cohen’s *d* for between-groups comparisons, Glass’s Δ for within-group comparisons) will be calculated. Nonparametric Analysis for Longitudinal Data (nparLD Package 2.1, R-software, v.3.6.1) will also be considered in cases where GEEs are not applicable (undefined distributions). If nparLD are used, effect sizes will be reported as relative treatment effect. Satisfaction with treatment will be compared between the two interventions using Chi-squared test for the PASS and Mann Whitney test or independent *t*-tests for satisfaction questions using the Likert scales. Exploratory analyses (using a similar statistical approach as for primary outcome) will be performed to compare the two interventions for each of the four conditions using the region-specific questionnaires (ODI, KOS-ADLS, *Quick*DASH, LEFS).

## Discussion

MSKd have a significant negative impact on military members’ operational readiness [11–13]. They lead to a high rate of days not available for duty and a high rate of personnel medically discharged from service [13, 14]. Furthermore, the capacity of the physiotherapy services has reached its limit compromising the management of MSKd in a timely manner, a mandatory criterion to maintain operational readiness [13, 14, 65]. This RCT will establish the effectiveness of group-based supervised training programs compared to usual individual physiotherapy care, which could provide an efficient, and more pro-active approach to manage a higher number of military members with musculoskeletal disorders. It could improve access to physical rehabilitation care and improve the health of military members by decreasing musculoskeletal pain and related functional limitations, as well as optimize operational readiness.

## Strengths and limitations

This RCT will be the first to investigate group-based intervention programs for various MSKd in militaries. It will provide knew knowledge to improve MSKd management in this population. In addition, to our knowledge, no studies have yet investigated group-based intervention programs for patellofemoral pain syndrome and lateral ankle sprains, and very few did for rotator cuff related shoulder pain. Thus, the results of this RCT will provide significant contribution to the literature. However, this study is a pragmatic trial and will be conducted in military population. Militaries are generally young, have daily trainings and have jobs with higher physical demands than the average population. Considering the specific (pragmatic) setting of this RCT and its population, the results of this RCT cannot extrapolated to other populations.

## Data Availability

There are ethical restrictions on sharing the de-identified data imposed by the ethics committee. Data will be made available on request from the ethics committee (lyne.martel2.ciussscn@ssss.gouv.qc.ca). If such request is made, the ethics committee will analyze the request (mainly look at the objectives behind the request) and then give a decision.

## Abbreviations

MSKd: Musculoskeletal disorders
PT: Physiotherapists
CAF: Canadian Armed Forces
UPC: Usual physiotherapy care
LBP: Low back pain
BPI: Brief Pain Inventory
TSK: Tampa scale of kinesiophobia
ODI: Oswesty Disability Index
QuickDASH: Shortened version of the Disability of the Arm, Shoulder and Hand
KOS-ADLS: Knee Outcome Survey of Activity of Daily Living Scale
LEFS: Lower Extremity Functional Scale
PASS: Patient Acceptable Symptom State
CID: Clinical Important Difference
MDC: Minimal Detectable Difference
GEE: Generalized Estimating Equations
